# The Insurance Bill and Charitable Institutions

**Published:** 1911-08-19

**Authors:** 


### HOSPITALS AND STATE INSURANCE.

### THE INSURANCE BILL AND CHARITABLE INSTITUTIONS.

In spite of the fact that the position of charitable institutions under the proposed National Insurance scheme has been frequently and fully discussed, the effect of the Bill on penitentiaries, homes for waifs and strays, homes for the feeble-minded, and similar institutions does not seem to be appreciated. The inmates of these institutions, although they do not receive wages, are employed persons within the meaning of the Insurance Bill, and the governors of the institutions will, unless the Bill is amended, be required to pay the employer's as well as the insurer's share—that is to say, 6d. a week in respect of females and 7d. in respect of males. Such inmates, however, will be excluded from sickness and disablement benefits so long as they remain in the institution, or, in other words, they will receive no adequate return for their subscriptions. Some amendment of the Bill designed to remedy this unsatisfactory position is obviously desirable, and it is satisfactory to note that the Chancellor of the Exchequer has consented to receive a deputation from the Charity Organisation Society regarding the inmates of these institutions. The best way of overcoming the difficulty no doubt would be to exclude the inmates of these institutions from the Bill altogether.

